# The Swedish version of OMAS is a reliable and valid outcome measure for patients with ankle fractures

**DOI:** 10.1186/1471-2474-14-109

**Published:** 2013-03-25

**Authors:** Gertrud M Nilsson, Magnus Eneroth, Charlotte S Ekdahl

**Affiliations:** 1 Department of Health Sciences, Division of Physical Therapy, Lund University, Lund, Sweden; 2 Department of Orthopaedics, Skane University Hospital, Lund University, Lund, Sweden

**Keywords:** Ankle fracture, OMAS, Reliability, Validity

## Abstract

**Background:**

The aim of this study was to evaluate the test-retest reliability and the validity of the self-reported questionnaire Olerud-Molander Ankle Score (OMAS) in subjects after an ankle fracture.

**Methods:**

When evaluating the test-retest reliability of the OMAS, 42 subjects surgically treated due to an ankle fracture participated 12 months after injury. OMAS was completed by the patients on two occasions at one to two weeks’ interval. Concurrent criterion validity was evaluated using the five subscales of the Foot and Ankle Outcome Score (FAOS) and global self-rating function (GSRF), which is a five-grade Likert scale with the alternatives: “very good”, “good”, “fair”, “poor”, “very poor”. Forty-six patients participated in the validation against FAOS, and for GSRF 105 patients participated at 6 months and 99 at 12 months. Uni-, bi- and trimalleolar fractures were all included and both non-rigid and rigid surgical techniques were used. All fractures healed without complications. Before analysis of the results the five groups according to GSRF were reduced to three: “good”, “fair” and “poor”. Test-retest reliability was assessed using Spearman’s rank correlation, the intraclass correlation coefficient (ICC), the standard error of measurement (SEM and SEM%) and the smallest real difference (SRD and SRD%). The Cronbach’s alpha score and validity versus FAOS was assessed using Spearman’s rank correlation and validity versus GSRF using the Kruskal-Wallis Test and the Mann–Whitney *U*-Test as ad hoc analyses.

**Results:**

The test-retest reliability correlation coefficient obtained was rho = 0.95 and ICC = 0.94. The SEM was 4.4 points and SEM% 5.8% and should be interpreted as the smallest change that indicates a real change of clinical interest for a group of subjects. The SRD was 12 points and SRD% 15.8% and should be interpreted as the smallest change that indicates a real change of clinical interest for a single subject. The correlation coefficients versus the five subscales of FAOS ranged from rho = 0.80 to 0.86. There were significant differences between GSRF groups “good”, “fair” and “poor” (p < 0.001) at both the six-month and the 12-month follow-up. The internal consistency for the OMAS was 0.76. The effect size between results from 6-month and 12-month follow-up turned out be 0.44 and should be considered as medium.

**Conclusion:**

The results showed that the test-retest reliability of the Swedish version of OMAS was very high in subjects after an ankle fracture and the standard error of measurement was low. Furthermore the OMAS was found to be valid using both the five subscales of FAOS and the GSRF. The OMAS can thus be used as an outcome measure after an ankle fracture.

## Background

Fractures involving the ankle are increasing [1] and are one of the most common fractures in the lower extremity [2,3] with an incidence rate of 101 fractures per 10^5^ person-years [3]. Ankle fractures occur in all ages and during different types of daily activities [4,5]. The age-adjusted incidence rate for the two genders has been reported as equal [4], but in younger ages the incidence rate is higher among men and at the age of 50 the gender ratio reverses [2,4]. The number of ankle fractures in the elderly is increasing [1,6] and in women over 65 years of age it has been found to be 300 per 10^5^ person-years [7]. Most fractures are surgically treated with open reduction and internal fixation due to dislocation [8-12]. After surgery the ankle is normally immobilized in a below knee plaster cast [13-15] or in a brace [16-18] for six to eight weeks.

Many investigators have evaluated both short- and long-term results after surgery [16,19-25]. Radiographic assessments [23,26], ankle mobility [16,22,26-29] and muscle strength [16,22,29,30] have been studied. It has however become more common to use patient-reported scores to evaluate functional results, as has been done by many authors [16,18,22,28,31]. As suggested by the International Classification of Functioning, Disability and Health (ICF), the degree of impairments, disabilities, participation problems and health related quality of life should be described from the patient’s perspective [32]. Patient-reported instruments such as questionnaires are appropriate instruments for this purpose.

Several scores have been developed in order to evaluate function after ankle injuries [26,33-35]. The Karlsson score [33] is a patient self-reported questionnaire while the Kaikkonnen score [34] has to be completed by both patient and clinician as six of the nine items consist of clinical tests. Both are mainly intended to evaluate function after ankle ligament injuries and both have been found to be valid [36]. The Foot and Ankle Outcome Score (FAOS) is a self-reported questionnaire and was developed to assess function in a variety of foot and ankle-related problems (http://www.koos.nu). FAOS has been found valid and reliable [35].

The Olerud-Molander Ankle Score (OMAS) is a disease-specific questionnaire devised for patients with ankle fractures and has been frequently used to evaluate subjectively scored function in this patient group [16,19,22,23,26,29,31,37]. OMAS has been validated against: Linear Analogue Scale (LAS) assessing subjective evaluation of ankle function on a 15 cm long linear analogue scale with the ends marked “perfectly normal ankle” and “totally disabling ankle” (p < 0.01), range of motion in dorsal extension (p < 0.05), presence of osteoarthritis grade II-IV (p < 0.001) and presence of dislocations on radiographs (p < 0.05) after an ankle fracture [26]. It has also been found to discriminate for subjectively experienced ankle instability (p < 0.02) and muscle strength in the ankle dorsi- and plantar flexors (p < 0.02) [22]. Wees et al. examined concurrent validity in patients with acute ankle ligament injuries and compared the Ankle Function Score (AFS) and the OMAS. The concurrent validity between the two scores at baseline and at follow-up was found to be good (r_p_ = 0.82 and 0.70) [38]. Furthermore OMAS has been reported capable of recording change over time in the short-term after an acute ankle ligament injury. The day seven effect size was reported to 1.3 and day 14 to 2.3 [36,39]. No floor or ceiling effects have been found when evaluating patients with acute ankle ligament injuries [38]. Although frequently used, few methodological studies regarding OMAS have been performed [22,26,38,39] and to the best of our knowledge no studies have been published assessing the reliability of the OMAS instrument [36], effect size in the long-term or validity using another disease-specific questionnaire or self-rated function with a graded rating scale in patients with surgically treated ankle fracture. The aim of this study was therefore to evaluate the test-retest reliability and the validity of the self-reported questionnaire OMAS in subjects after an ankle fracture.

## Methods

### Design and participants

This is a test-retest reliability study and a concurrent validity study of OMAS. All participants were from the same cohort and were recruited at the University Hospital in Lund, Sweden. This study is a part of an earlier intervention study [29] which was approved by the Research Ethics Committee at Lund University, Lund, Sweden. Written informed consent had to be provided for participation in the study.

### Test-retest reliability of OMAS

#### Participants

Forty-two subjects, 23 women and 19 men surgically treated due to an ankle fracture, participated in the evaluation of the test-retest reliability. The mean age was 42 (SD 14), 23 had a unimalleolar fracture and 19 had a bi- or trimalleolar fracture. Both non-rigid and rigid surgical techniques had been used. Fracture types and surgical techniques that were used have been described in detail elsewhere [29]. All subjects had been immobilized non-weight-bearing in a below-knee plaster cast and the plaster time was mean 43 days (SD 5.5). All fractures healed without complications.

#### Test procedure

The test-retest reliability of OMAS was studied 12 months after injury. The questionnaire was completed on two separate occasions. The first time OMAS was filled in at home by the 42 subjects and then sent by mail to the test leader (GN). The second time OMAS was filled in at the clinic when the subjects were called for a physical examination as part of a follow-up study [29]. The two sessions were spaced one to two weeks apart. The subjects did not have access to the first version when the second was filled in.

### Validity of OMAS using global self-rated function

#### Participants

One-hundred-five patients, 63 women and 42 men, participated in the validation of the OMAS at 6 months. The mean age was 44 (SD 14); 65 subjects had a unimalleolar fracture and 40 had a bi- or trimalleolar. At 12 months, 99 patients, 59 women and 40 men participated, the mean age was 45 (SD 14) and 64 had a unimalleolar and 35 bi- or trimalleolar fracture. Surgical techniques and immobilization routines were the same as described above.

#### Test procedure

OMAS and the global self-rated function (GSRF) were filled in at home six and twelve months after the injury and then sent by mail to the test leader (GN).

### Validity of OMAS using the Foot and Ankle Outcome Score

#### Participants

Forty-six patients, 26 women and 20 men, participated in the validation of OMAS versus the Foot and Ankle Outcome Score (FAOS). The mean age was 43 (SD 14) and 25 had a unimalleolar fracture and 21 a bi- or trimalleolar. Surgical techniques and immobilization routines were the same as described above.

#### Test procedure

OMAS was filled in 12 months after the injury and sent by mail to the test leader. FAOS was filled in at the clinic when the subjects were called for a physical examination as part of a follow-up study [29].

### Outcome measures

#### OMAS

OMAS is a self-administered patient questionnaire [26]. The scale is an ordinal rating scale from 0 points (totally impaired function) to 100 points (completely unimpaired function) and is based on nine different items given different points: pain (0–25), stiffness (0–10), swelling (0–10), stair climbing (0–10), running (0–5), jumping (0–5), squatting (0–5), supports (0–10) and work/activity level (0–20). The different symptoms are stated and have different points according to the extent of disability the authors considered they would lead to [26]. The score is calculated as the sum of each rated item. The original version of the OMAS was created in Swedish and this version was the subject of the present study. Some minor changes were made in the version that was tested in the present study. In item three, dealing with swelling, the response alternatives in the original version were “none”, “only evenings” and “constant”. In the version that we tested the alternatives were “none”, “after overuse or only during the evening” and “constant”. In item four, dealing with stair-climbing, the response alternatives in the original version were “no problems”, “impaired” and “impossible”. In the version that we tested the item was changed to “stairs” instead of “stair climbing” and the response alternatives were “no problems”, “some problems” and “impossible”. These changes were made with permission from the Swedish developer, Professor Claes Olerud (16 March 2012).

#### GSRF

The GSRF is a self-administered ordinal five-grade rating scale. The patients have to evaluate their present ankle function using five alternatives: “very good”, “good”, “fair”, “poor” and “very poor”.

Global self-rated ankle function can be assessed, for example, by LAS [17,22,26,33] or by a five-grade Likert scale (excellent, good, fair, poor, and very poor).

#### FAOS

FAOS is also a self-administered patient questionnaire and consists of 42 items divided into five subscales: pain (9 items), other symptoms (7 items), function in daily living (ADL) (17 items), function in sport and recreation and foot (4 items) and ankle-related quality of life (5 items). Standardized options are given and for each item a five-point Likert scale is used (no, mild, moderate, severe, extreme). Each item gets a score from 0–4 and each of the five subscale scores is calculated as the sum of the rated items included. Raw scores are then transformed to a scale 0 (indicating extreme symptoms) to 100 (indicating no symptoms) (http://www.koos.nu).

FAOS, developed from the self-reported questionnaire KOOS (Knee Injury and Osteoarthritis Outcome Score), has been found to be reliable over time in subjects with surgically treated ankle ligament injuries [35] and valid against three subscales of SF-36 (bodily pain, physical functioning and social functioning) (p < 0.01) in subjects with different foot and ankle disorders in the Turkish version of FAOS [40]. Furthermore, it has been found valid against the patient-reported instrument Achilles Tendon Total Rupture Score (ATRS) in subjects with a total achilles tendon rupture. All subscales of FAOS correlated well with that instrument (p < 0.01) [41].

### Statistics

Statistical analyses were performed using the SPSS software version 17.0. As OMAS is an ordinal scale non-parametric statistics have been used. However, to be able to analyze the standard error of measure (SEM), the smallest real difference (SRD) and effect size (ES) also the mean values and standard deviations a had to be applied. All correlation coefficients (rho) were calculated using Spearman’s rank correlation, with a coefficient level of < 0.5 considered as low, 0.5–0.69 as moderate, 0.7–0.89 as high and 0.9–1.0 very high [42]. To analyze the agreement between the two repeated measurements at 12-month the intraclass correlation coefficient (ICC) was applied. To check for systematic error between the two measurements, the Wilcoxon’s signed rank test was used. Internal consistency of OMAS was calculated using Cronbach’s alpha [43]. The standard error of measure (SEM) was defined by SEM = SD √(1-ICC) and SEM% by (SEM/mean) × 100 where mean is the mean for all values from test session 1 and 2. The smallest real difference (SRD) was defined by SRD = 1.96 × SEM × √2 and the SRD% by SRD/mean × 100 where mean is the mean for all values from test session 1 and 2. An ‘error band’ around the mean difference of the two measurements, *d*, was defined by 95% SRD = *d ±* SRD [44]. Effect size of OMAS between six-month and twelve-month follow-up was calculated as (mean value of measurement 2 – mean value of measurement 1)/SD of measurement 1 [45]. Significance was considered at the alpha level of p < 0.05. Before the statistical analysis of the validity of OMAS versus the five-grade rating scale, the subjects were reduced to three groups. Those who had answered “very good” and “good” formed one group (Group 1), “fair” formed one group (Group 2) and those who had answered “poor” and “very poor” formed one group (Group 3). When comparing the results between the three groups the Kruskal Wallis test was used and as ad hoc between each group the Mann–Whitney *U*-test was applied.

## Results

### Test-retest reliability and internal consistency

No significant differences were found between the two measurements of OMAS (p = 0.14) and the correlation (rho = 0.95) and agreement (ICC = 0.94) were both very high (Table 1). The standard error of measurement (SEM) which represents the smallest change that indicates a real (clinical) improvement or worsening for a group of subjects was 4.4 points and SEM% was 5.8%. The smallest real difference (SRD) which is the equivalence for a single subject was 12.0 points and the SRD% was found to be 15.8%. The 95% SRD which represents the limits for the smallest change for a single subject ranged from −10.4–13.7. The internal consistency of the 9 items was 0.76 as calculated with Cronbach’s alpha.

**Table 1:** Values of OMAS at first and second measurements and correlation between the two measurements

**Variable**	**First measurement**	**Second measurement**	**p-value**	**Correlation**	**p-value correlation**
	**n = 42**	**n = 42**			
OMAS median (range) (IQR)	78 (30–100) (26)	80 (35–100) (30)	p = 0.14¤	rho =0.95	p < 0.001
OMAS mean (SD)	75 (19)	77 (18)		ICC = 0.94	p < 0.001

¤Wilcoxon sign rank test; rho_=_ spearman correlation coefficient; *IQR*, Interquartile range; *ICC*, intraclass correlation coefficient.

### Validity of OMAS versus GSRF

There were significant differences in the scoring rates of OMAS between the three groups of GSRF at both the six-month (Table 2) and the 12-month follow-up (Table 3).

**Table 2:** OMAS versus global self-rated function six months after injury

**Variable**	**Group 1**	**Group 2**	**Group 3**	**p-value**
	**good**	**fair**	**poor**	
	**n = 44**	**n = 36**	**n = 25**	
OMAS 6-month median (range) (IQR)	80 (0–100) (19)	60 (20–85) (20)	40 (10–60) (25)	p < 0.001¤

¤Kruskal-Wallis Test.

Ad hoc analysis: Group 1 vs Group 2 = p < 0.001 (Mann–Whitney *U*-test).

Ad hoc analysis: Group 2 vs Group 3 = p < 0.001 (Mann–Whitney *U*-test).

**Table 3:** OMAS versus global self-rated function 12 months after injury

**Variable**	**Group 1**	**Group 2**	**Group 3**	**p-value**
	**good**	**fair**	**poor**	
	**n = 58**	**n = 26**	**n = 12**	
OMAS 12-month median (range) (IQR)	85 (50–100) (25)	60 (30–75) (25)	45 (15–75) (19)	< 0.001¤

¤Kruskal-Wallis Test.

Ad hoc analysis: Group 1 vs Group 2 = p < 0.001 (Mann–Whitney *U*-test).

Ad hoc analysis: Group 2 vs Group 3 = p = 0.021 (Mann–Whitney *U*-test).

### Validity of OMAS versus FAOS

The correlation between OMAS and the five subscales of FAOS was high. All correlation coefficients reached or exceeded 0.8 (Table 4).

**Table 4:** Validity of OMAS versus the five subscales of the foot and ankle outcome score

**OMAS median (range) (IQR)**	**FAOS subscales median (range) (IQR)**	**Spearman’s correlation coefficient (rho)**	**p-value correlation**
**n = 45**	**n = 45**		
80 (30–100) (25)	Pain 92 (47–100) (22)	0.86	<0.001
80 (30–100) (25)	Symptoms 79 (30–100) (37)	0.81	<0.001
80 (30–100) (25)	ADL 99 (54–100) (9)	0.80	<0.001
80 (30–100) (25)	Sports 80 (0–100) (45)	0.85	<0.001
80 (30–100) (25)	QoL 75 (25–100) (26)	0.81	<0.001

*IQR*, Interquartile range.

### Score distribution and effect size

OMAS varied from 0–100 at six months and 15–100 at 12 months. One person scored 0 at six months. Six persons (5%) scored 100 at six months and 18 persons (15%) scored 100 at 12 months. Effect size turned out to be 0.44 calculated as (73.04-62.99)/23.

## Discussion

The main results of this study showed that the test-retest reliability and concurrent validity of the OMAS were good for patients surgically treated due to an uni- bi or trimalleolar ankle fracture.

Reliability is an important dimension of any patient-based outcome measure as it is essential to establish whether changes observed are due to the intervention and not to variations related to problems with the outcome instrument. The larger the random error, the larger sample size is needed in order to obtain precise estimates of effects in a trial [45]. There are two aspects that have to be considered when evaluating reliability: reproducibility and internal consistency. A correlation coefficient in a test-retest of a measurement tool should be at least 0.70 when studying groups of patients and exceed 0.9 when studying individuals [45,46]. Internal consistency can be evaluated using Cronbach’s alpha (0–1) [43]. When items are used to form a scale they should have internal consistency, which means they should measure the same thing and be correlated to each other. When comparing groups an alpha level of 0.7–0.8 is recommended [43,45]. Too high levels of alpha indicate that all items are identical, addressing a rather narrow aspect of an attribute [43] or there is redundancy among items [45]. Too low levels indicate that the items included in the scale are not related to each other [43].

To the best of our knowledge no previous studies have evaluated the test- retest reliability of OMAS in subjects surgically treated due to an ankle fracture. Both the Spearman’s rank correlation coefficient and the ICC between the two measurements turned out to be very high (rho =0.95 and ICC = 0.94), although the circumstances of the two occasions when the questionnaires were filled in were not exactly the same; the first version was filled in at home and the second at the clinic. Cronbach’s alpha was within the recommended limits (0.76) and thus the OMAS can be regarded as a reliable measure in subjects after an ankle fracture.

Even if an ICC value is high it does not mean that a test is appropriate for clinical use. Before recommending a test for that issue the measurement errors both for groups of subjects and for single individuals have to be analyzed as well. The measurement error of an instrument should be small and sensitive enough to detect real changes in scored function. In the present study the SEM and SRD were used which gave the measurement errors in absolute values. SEM was 4.4 points and SEM% was 5.8%. These figures are both low and should be regarded as indicative for true change beyond measurement error and applied as the smallest difference between two measurements when evaluating for example an intervention on group level. The 95% SRD ranged from −10–13.7 points and indicates that a real clinical change for a single subject should exceed this range. The SRD% is independent of the unit of measurement like SEM% and may be more easy to use in clinical practice. The SRD% found was 15.8% and thus if a subject scores for example 60 points this subject must improve 10 points to indicate a real change. From a clinical point of view these values seem to be reasonable and confirm that OMAS can be used to detect real changes in subjects after an ankle fracture.

Criterion validity is the extent to which one measure is related to other measures or outcomes. This type of validity can be divided into either concurrent or predictive validity; concurrent validity is when a new tool is to be compared at the same time with another measurement as gold standard [47]. Often, however, a true gold standard against which a new measurement can be compared does not exist, and this was the case in our study. In the present study OMAS was validated using two different instruments, the disease-specific questionnaire FAOS and the global rating scale, GSRF. FAOS has been tested for reliability and validity in the Swedish version [35], the Turkish version [40] and the Iranian version [48]. The ICC values for test-retest reliability were high [40,48] and the validity using SF-36 varied between low and moderate in the Iranian version [48] and between low and high in the Turkish version [40], but in that study only three subscales of SF-36 were presented. SF-36 is an instrument evaluating generic health-related quality of life, including equal parts of mental health and physical health, whereas FAOS is a disease-specific instrument evaluating functional outcome of the ankle. Thus not all subscales of SF-36 might be expected to correlate well with FAOS.

We found that the correlations between OMAS and the five subscales of FAOS were all high. When looking at the figures it can be noticed that for the subscale ADL in FAOS the median value was 99 and the interquartile range was nine. These figures are more extreme than they are for the rest of the items, and it seems as if the suggested functions were minor problems for the patients included in our study. FAOS has been developed from the self-reported questionnaire KOOS aimed at evaluating function in subjects with osteoarthritis in the knee, and the questions are adjusted and relevant to those patients. We think that not all items might be suitable for patients after an ankle fracture especially, not in the ADL subscale where many items deal with problems in non-weight-bearing positions such as “bending to floor”, “putting on socks”, “taking off socks”, “lying in bed”. It is logical to believe that if patients do not identify with the problems presented, the total score of those items would be rated higher. In the validation study of FAOS the authors came to the same conclusion; only two of 17 items were considered as “at least of some importance” by the responders [35]. Regarding the subscale pain, the correlation was high but again the numerical value for FAOS was higher, and again many of the items of that subscale of FAOS deal with pain in non-weight-bearing positions such as “at night while in bed”, “sitting or lying”, “bending foot/ankle fully”, “stretching foot/ankle fully”. These situations are probably a minor problem in the studied group with ankle fractures.

Global rating of function was the traditional way of presenting results after treatment of injuries or diseases in earlier studies [8,11,49]. As a clinician it is important to take into account the patient’s overall opinion about recovery and function after an injury. It should be optimal if the returned responses from a disease-specific instrument agree with the patient’s overall rated function from the same region. OMAS has been shown to correlate well in that respect using LAS [26]. In the present study the evaluation using GSRF agreed well with results from OMAS. The points from the three groups “good”, “fair” and “poor” were significantly separated at both the six-month and the 12-month follow-up.

Responsiveness is one dimension of great importance to determine when evaluating the methodological quality of a measurement. The responsiveness of an instrument expresses its capability to detect changes over time or changes due to intervention. In the present study the responsiveness was evaluated by calculating the effect size and was found to be 0.44 which could be considered as medium [45]. In the study by Rose et al. the effect size was evaluated in the early stages after an acute ankle ligament injury and was found to range from 1.3 to 2.3 [39]. It is well known that ankle ligament injuries recover quickly during the first two weeks, thereby the figures between that group and the group that we have focused on should differ. Between six and 12 months after an ankle fracture the improvement can be expected to progress more slowly and by then the effect size found should be regarded as realistic. There is a lack of knowledge how the responsiveness of OMAS is in the early phases of the rehabilitation process after ankle fractures and further studies regarding this are required.

However, to be able to detect changes over time the floor and ceiling effects should be considered as well, which means the number of patients reporting the lowest score at baseline should be limited in order to be able to observe deterioration. In the same way ceiling effect occurs when a patient reports excellent function and receives the best possible score. Questionnaires with good validity are expected to have fewer categories with floor or ceiling effects and no more than 15% of the individuals should score on these levels [40]. In the present study no floor or ceiling effects regarding OMAS were found at six months. At 12 months 15% scored 100, which is within the recommended limits, and one year after injury it can be expected that some of the patients have attained normal function.

OMAS is well known and has been used in lots of studies for several years [16,17,19,22,23,26,28,29],[37,50]. With relatively few items it can be easily completed and the items included are all relevant to normal activities of daily life. The score is simple for the researcher to use as the raw score is summed up without any further calculation. The different symptoms are given and have different points according to the extent of disability the authors considered they would lead to [26]. It seems as if these differences are reasonable, as OMAS and self-reported global function using both LAS [26] and GSRF in the present study have shown good correlations. Pain during different weight-bearing situations is the item that is highest weighted. It seems relevant as the foot and ankle take weight during every step all day long and pain in these situations should be strongly disabling. In two of our earlier studies we found that one year after the injury more than half of the patients still experienced pain while walking [22,31].

Ankle fractures surgically treated normally need stabilization of the ankle mortise using either staples or a screw [8,9,11] and immobilization for six to eight weeks after surgery in a plaster cast or in an orthosis [51]. These treatments influence the mobility of the ankle. The surgery technique might diminish the flexibility in the mortise and perhaps also the range of motion in dorsiflexion in the ankle joint. Maximum of dorsiflexion is needed during many activities of daily living such as climbing on a stool, rising from a chair, walking downstairs, walking uphill, rising from floor etc. Problems in those situations remind the patient of the injury and would thus be reflected in the score. Many authors have reported that patients still complained about stiffness in the ankle one year after the injury [16,17,22,31].

Swelling has been frequently reported in the studied patient group [15,17,22,31]. Swelling probably also affects the experience of stiffness and pain. Both stiffness and swelling are graded relatively high in the scoring scales, which seems relevant. Running and jumping are weighted lower. Ankle fracture occurs in all ages but increases with higher age [1,6], particularly in women [7]. Most ankle fractures happen to persons when stumbling or slipping in everyday life rather than to athletes [4]. It is thus likely that these functions have less influence in the main group of injured persons, which might be the reason why these items were weighted lower by the authors [26]. However, in younger age groups these functions are probably more important to regain, and then the weighting could be discussed. Furthermore, impediments or inabilities in return to work or to earlier activities of daily life might have a great impact on a person’s life. This item is weighted high, which seems to be correct.

Despite having relatively few items, the OMAS includes all dimensions of ICF recommended by the WHO [32]. Pain, stiffness and swelling belong to the domain Body function; stair-climbing, jumping, running and squatting to the domain Activity; and work/activities of daily life to the domain Participation. OMAS thus also fulfills these demands.

## Conclusion

In conclusion, the results of this study showed that the test-retest reliability of the Swedish version of OMAS was very high in subjects surgically treated after an ankle fracture and the standard error of measure was low. Furthermore, the concurrent validity using FAOS and GSRF was high. OMAS can thus be used as an outcome measure after an ankle fracture.

## Competing interests

The authors declare they have no competing interests.

## Authors’ contribution

GN participated in the design of the study, collected the data, performed the statistical analyses, and drafted the manuscript. ME was responsible for identifying and including the patients, and participated in the progress and revision of the manuscript. CE participated in the design of the study, and the progress and revision of the manuscript. All authors read and approved the final manuscript.

## Pre-publication history

The pre-publication history for this paper can be accessed here:

http://www.biomedcentral.com/1471-2474/14/109/prepub

## References

[B1] Kannus P Palvanen M Niemi S Increasing number and incidence of low-trauma ankle fractures in elderly people: Finnish statistics during 1970–2000 and projections for the future Bone 2002 31 430 433 10.1016/S8756-3282(02)00832-3 12231418

[B2] Van Staa TP Dennison EM Leufkens HG Epidemiology of fractures in England and Wales Bone 2001 29 517 522 10.1016/S8756-3282(01)00614-7 11728921

[B3] Court-Brown C Caesar B Epidemiology of adult fractures: A review Injury 2006 37 691 697 10.1016/j.injury.2006.04.130 16814787

[B4] Jensen S Andresen B Mencke S Epidemiology of ankle fractures. A prospective population-based study of 212 cases in Aalborg, Denmark Acta Orthop Scand 1998 69 48 50 10.3109/17453679809002356 9524518

[B5] Bischoff-Ferrari HA Orav JE Barrett J Effect of seasonality and weather on fracture risk in individuals 65 years and older Osteoporos Int 2007 18 1225 1233 10.1007/s00198-007-0364-6 17384897

[B6] Kannus P Parkkari J Niemi S Epidemiology of osteoporotic ankle fractures in elderly persons in Finland Ann Int Med 1996 125 975 978 8967708 10.7326/0003-4819-125-12-199612150-00007

[B7] Hasselman C Molly T Stone K Foot and ankle fractures in elderly white women. Incidence and risk factors J Bone Joint Surg Am 2003 85-A 820 824 12728031 10.2106/00004623-200305000-00008

[B8] Cedell C-A Supination-outward rotation injuries of the ankle Acta Orthopaedica Scandinavia, Suppl No. 110 1967 Copenhagen: Munksgaard 10.3109/ort.1967.38.suppl-110.01 4970319

[B9] Pettrone F Gail M Pee D Quantitative criteria for prediction of the results after displaced fracture of the ankle J Bone Joint Surg Am 1983 65 667 677 6406511

[B10] De Souza L Gustilo R Meyer T Results of operative treatment of displaced external rotation-abduction fractures of the ankle J Bone Joint Surg Am 1985 67 1066 1074 3928632

[B11] Lindsjö U Operative treatment of ankle fracture-dislocations: a follow-up of 306/321 consecutive cases Clin Orthop 1985 199 28 38 3930122

[B12] Stufkens SA van den Bekerom MP Doornberg JN Evidence-Based Treatment of Maisonneuve Fractures J Foot Ankle Surg 2011 50 62 67 Review 10.1053/j.jfas.2010.08.017 21172642

[B13] Lindsjö U Operative treatment of ankle fractures. [In Swedish: Operativ behandling av fotledsfrakturer] PhD thesis 1980 Uppsala, Sweden: Uppsala University

[B14] Olerud C Molander H Bi- and trimalleolar ankle fractures operated on with nonrigid internal fixation Clin Orthop Relate Res 1986 206 253 260 3086009

[B15] Ahl T Dalen N Selvik G Ankle fractures. A clinical and roentgenographic stereophotogrammetric study Clinical Orthop Relate Res 1989 245 246 255 2502344

[B16] Tropp H Norlin R Ankle performance after ankle fracture: a randomized study of early mobilization Foot Ankle Int 1995 16 79 83 10.1177/107110079501600205 7767451

[B17] Hedström M Ahl T Dalen N Early postoperative ankle exercise. A study of postoperative lateral malleolar fractures Clinical Orthop Relate Res 1994 300 193 196 8131334

[B18] Lehtonen H Jarvinen TL Honkonen S Use of a cast compared with a functional ankle brace after operative treatment of an ankle fracture. A prospective, randomized study J Bone Joint Surg Am 2003 85-A 205 211 12571295 10.2106/00004623-200302000-00004

[B19] Belcher GL Radomisli TE Abate JA Functional outcome analysis of operatively treated malleolar fractures J Orthop Trauma 1997 11 106 109 10.1097/00005131-199702000-00007 9057145

[B20] Egol KA Dolan R Koval KJ Functional outcome of surgery for fractures of the ankle. A prospective, randomised comparison of management in a cast or a functional brace J Bone Joint Surg Br 2000 82 46 49 10755435

[B21] Egol KA Tejwani NC Walsh MG Predictors of short-term functional outcome following ankle fracture surgery J Bone Joint Surg Am 2006 88 974 979 10.2106/JBJS.E.00343 16651571

[B22] Nilsson G Nyberg P Ekdahl C Performance after surgical treatment of patients with ankle fractures 14-month follow up Phys Res Int 2003 8 69 82 10.1002/pri.274 12879729

[B23] Lash N Horne G Fielden J Ankle fractures: functional and lifestyle outcomes at 2 years ANZ J Surg 2002 72 724 730 10.1046/j.1445-2197.2002.02530.x 12534384

[B24] Day G Swanson C Hulcombe B Operative treatment of ankle fractures: a minimum ten-year follow-up Foot Ankle 2001 2 102 106 11249218 10.1177/107110070102200204

[B25] Bauer M Ankle fractures. With special reference to post-traumatic arthrosis PhD thesis 1985 Lund, Sweden: Lund University

[B26] Olerud C Molander H A scoring scale for symptom evaluation after ankle fracture Arch Orthop Trauma Surg 1984 103 190 194 10.1007/BF00435553 6437370

[B27] Ahl T Dalen N Lundberg A Bylund C Early mobilization of operated on ankle fractures. Prospective, controlled study of 40 bimalleolar cases Acta Orthop Scand 1993 64 95 99 10.3109/17453679308994541 8451961

[B28] Ponzer S Nåsell H Bergman B Functional outcome and quality of life patients with Type B ankle fractures: a two-year follow up study J Orthop Trauma 1999 13 363 368 10.1097/00005131-199906000-00007 10406704

[B29] Nilsson GM Jonsson K Ekdahl CS Effects of a training program after surgically treated ankle fracture: a prospective randomised controlled trial BMC Musculoskelet Disord 2009 10 118 10.1186/1471-2474-10-118 19781053 PMC2760502

[B30] Shaffer M Okereke E Esterhai J Effects of immobilization on plantar-flexion torque, fatigue resistance, and functional ability following an ankle fracture Phys Ther 2000 80 769 780 10911415

[B31] Nilsson GM Jonsson K Ekdahl CS Outcome and quality of life after surgically treated ankle fractures in patients 65 years or older BMC Musculoskelet Disord 2007 20 8 127 18096062 10.1186/1471-2474-8-127 PMC2259334

[B32] WHO ICF – international classification of functioning, disability and health 2001 Geneva: WHO Library

[B33] Karlsson J Peterson L Evaluation of ankle and joint function: the use of a scoring scale The Foot 1991 1 15 19 10.1016/0958-2592(91)90006-W

[B34] Kaikkonen A Kannus P Järvinen M A performance test protocol and scoring scale for the evaluation of ankle injuries Am J Sports Med 1994 22 462 469 10.1177/036354659402200405 7943510

[B35] Roos EM Brandsson S Karlsson J Validation of the foot and ankle outcome score for ankle ligament reconstruction Foot Ankle Int 2001 22 788 794 11642530 10.1177/107110070102201004

[B36] Haywood KL Hargreaves J Lamb SE Multi-item outcome measures for lateral ligament injury of the ankle: a structured review J Eval Clin Pact 2004 10 339 352 10.1111/j.1365-2753.2003.00435.x 15189400

[B37] Wang R Thur CK Gutierrez-Farewik EM One year follow-up after operative ankle fractures: a prospective gait analysis study with a multi-segment foot model Gait Posture 2010 31 234 240 10.1016/j.gaitpost.2009.10.012 19942435

[B38] Wees P Hendriks E Beers H Validity and responsiveness of the ankle function score after acute ankle injury Scand J Med Sci Sports 2012 22 170 174 10.1111/j.1600-0838.2010.01243.x 21083768

[B39] Rose A Lee RJ Williams RM Functional instability in non-contact ankle ligament injuries Br J Sports Med 2000 34 352 358 10.1136/bjsm.34.5.352 11049145 PMC1756237

[B40] Karatepe AG Günaydin R Kayak T Validation of the Turkish version of the foot and ankle outcome score Rheumatol Int 2009 30 169 173 10.1007/s00296-009-0929-0 19370349

[B41] Nilsson-Helander K Thomée R Silbernagel KG The achilles tendon total rupture score (ATRS) Am J Sports Med 2007 35 421 426 17158277 10.1177/0363546506294856

[B42] Munro BH Statistical Methods for Health Care Research 2000 4 JB Lippincott: Philadelphia. Pa

[B43] Bland JM Altman DG Cronbach’s alpha BMJ 1997 314 572 10.1136/bmj.314.7080.572 9055718 PMC2126061

[B44] Beckerman H Roebroeck ME Lankhorst GJ Smallest real difference, a link beteewen reproducibility and responsiveness Oual Life Res 2001 10 571 578 10.1023/A:1013138911638 11822790

[B45] Fitzpatrick R Davey C Buxton MJ Evaluation patient-based outcome measures for use in clinical trials Health Technol Assessment 1998 2 14 9812244

[B46] Nunnaly J Bernstein JC Psychometric theory 1994 3 New York: McGraw-Hill

[B47] Domholdt E Rehabilitation Research. Principles and Applications 2005 3 St Louis, Mo: Elsevier Saunders

[B48] Negahban H Mazaheri M Salavati M Reliability and validity of the foot and ankle outcome score: a validation study from Iran Clin Rheumatol 2010 29 479 486 10.1007/s10067-009-1344-3 20140474

[B49] Sefton GK George J Fitton JM McMullen H Reconstruction of the anterior talofibular ligament for the treatment of the unstable ankle J Bone Joint Surg Br 1979 61 352 354 113415 10.1302/0301-620X.61B3.113415

[B50] Nilsson GM Jonsson K Ekdahl CS Unsatisfactory outcome following surgical intervention of ankle fractures Foot Ankle Surg 2005 11 11 16 10.1016/j.fas.2004.10.004

[B51] Thomas G Whalley H Modi C Early mobilization of operatively fixed ankle fractures: a systematic review Foot Ankle Int 2009 30 666 674 Review 10.3113/FAI.2009.0666 19589314

